# Supplementary figures and images for: The DEAD-box RNA helicase Dhx15 controls glycolysis and arbovirus replication in Aedes aegypti mosquito cells
Source: PLoS Pathog. 2022 Nov 28;18(11):e1010694. doi: 10.1371/journal.ppat.1010694 (PMC9731432; doi:10.1371/journal.ppat.1010694)

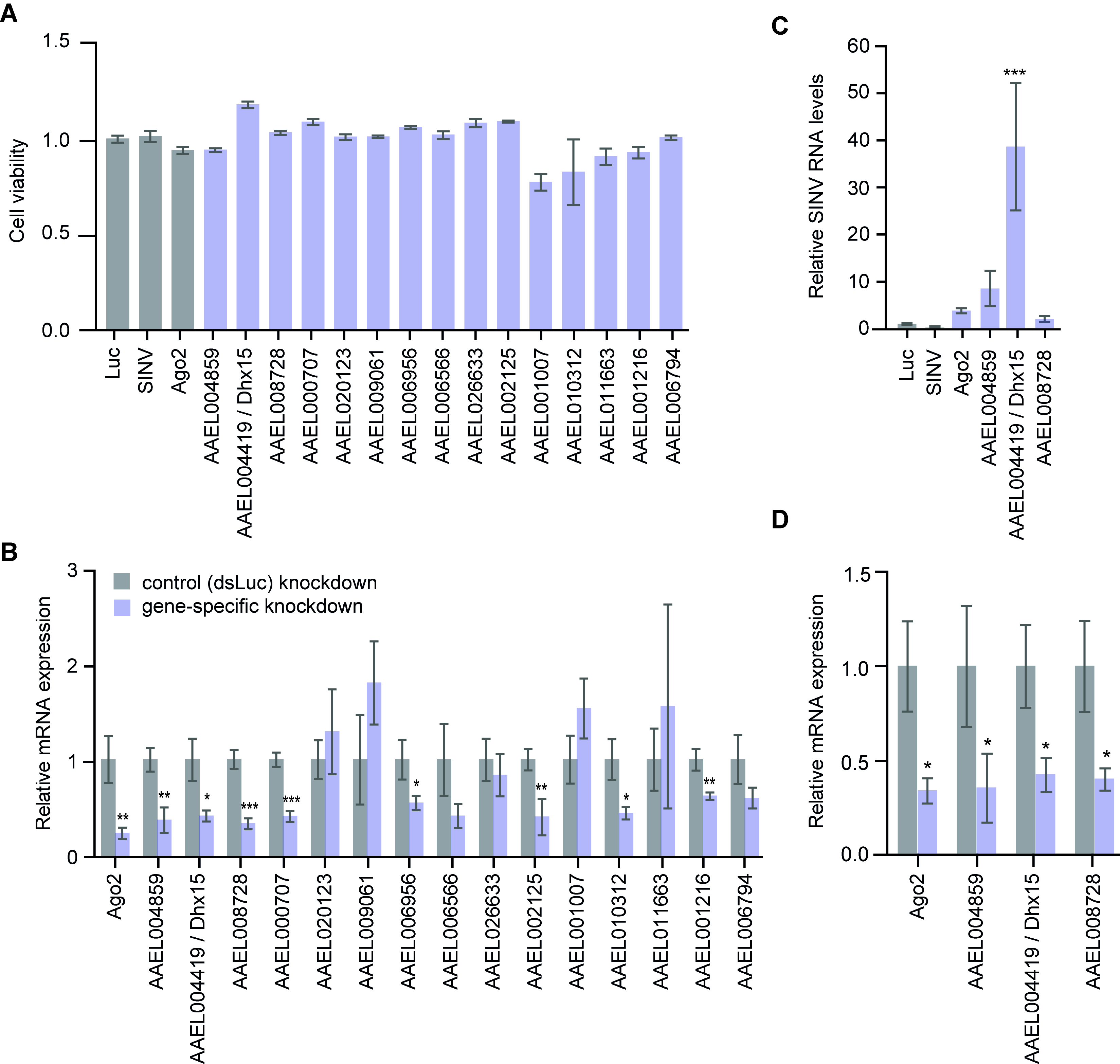

Supplement: S1 Fig — A) Viability of Aag2 cells was measured using CellTiter-Glo assay after silencing of 15 candidate genes (see Fig 1D and 1E) using the first set of dsRNA. Bars and whiskers represent the mean +/- SD of three independent biological replicates. B) Knockdown efficiency of 15 candidate genes (from the experiment shown in Fig 1E) was assessed by RT-qPCR. Bars and whiskers represent the mean +/- SD of three independent biological replicates. Statistical significance was determined using unpaired two tailed t-test (* p < 0.05, ** p < 0.005, *** p < 0.0005). C) Levels of SINV were quantified using RT-qPCR after silencing of Dhx15, AAEL008728, and AAEL004859 in Aag2 C3PC12 cells. SINV infection was performed with an MOI of 0.1. Bars and whiskers represent the mean +/- SD of three independent biological replicates. Statistical significance was determined using One-Way ANOVA with Holm-Sidak correction (*** p < 0.0005). D) Knockdown efficiency of genes from panel (C) were assessed by RT-qPCR. For each gene, the specific knockdown (light purple) was compared to dsLuc control knockdown (gray). Bars and whiskers represent the mean +/- SD of three independent biological replicates. Statistical significance was determined using unpaired two tailed t-test (* p < 0.05). (TIF) [file ppat.1010694.s001.tif]

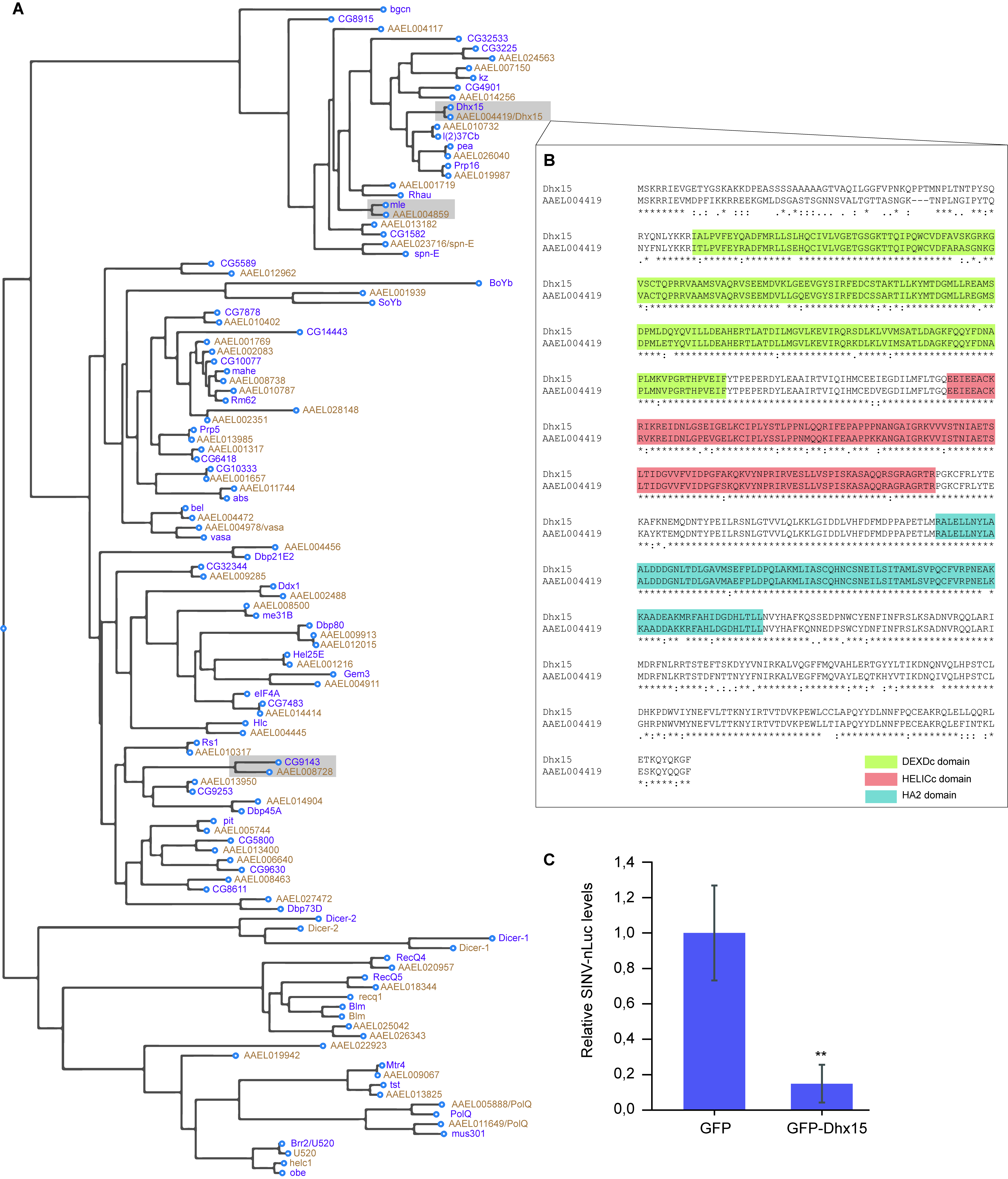

Supplement: S2 Fig — A) Unrooted approximately-maximum likelihood tree of Drosophila (purple) and Ae. aegypti (brown) RNA-helicases with branch lengths estimated using the CAT approximation described in [50]. B) Multiple sequence alignment of Drosophila Dhx15 and Ae. aegypti AAEL004419. The functional domains (see Fig 2A) are highlighted with colored boxes. C) SINV nLuc reporter expression was assessed in Aag2 C3PC12 cells upon transgenic expression of GFP tagged Dhx15. Cells transfected with an empty GFP vector served as negative control. Bars and whiskers show the mean +/- SD of three biological replicates. Statistical significance was determined using unpaired two tailed t-test (** p < 0.01). (TIF) [file ppat.1010694.s002.tif]

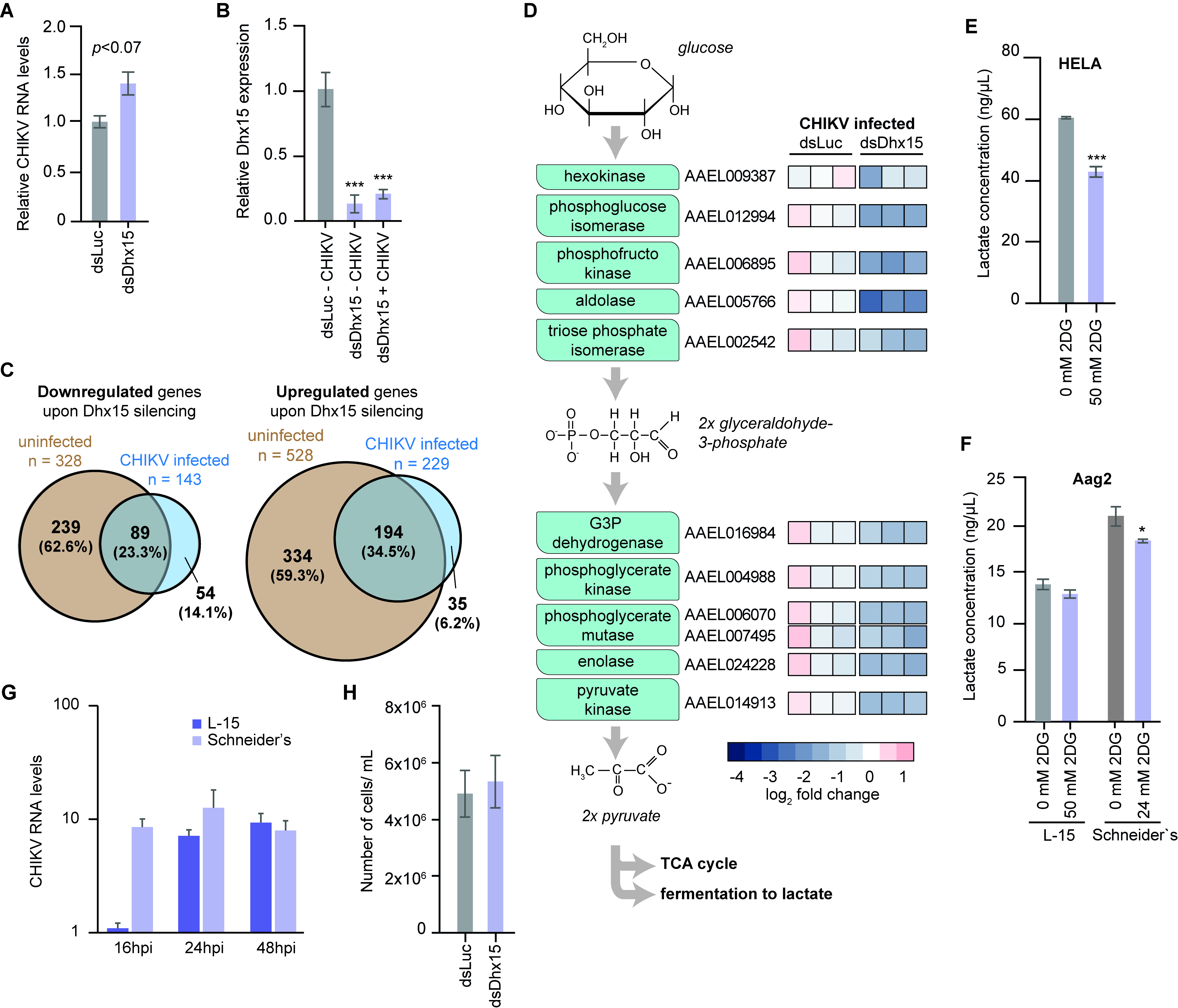

Supplement: S3 Fig — A-B) Levels of CHIKV (A) and knockdown efficiency of Dhx15 (B) in samples used for deep-sequencing were assessed by RT-qPCR. CHIKV infection was performed with an MOI of 5. Bars and whiskers represent the mean +/- SD of three independent biological replicates. Statistical significance was determined using unpaired two tailed t-tests (*** p < 0.0005). C) Number of overlapping genes downregulated (left panel) and upregulated (right panel) upon Dhx15 silencing in uninfected and CHIKV infected cells. D) Schematic representation of the enzymes involved in the glycolysis pathway (left) and log2 fold change of these genes upon Dhx15 or firefly luciferase silencing (right) in CHIKV infected cells. E-F) Relative lactate concentration upon 2-deoxy-D-glucose (2-DG) treatment in Hela (E) and Aag2 (F) cells. G) CHIKV RNA levels in Aag2 C3PC12 cells infected at an MOI of 0.1 and cultured in L-15 and Schneider’s medium, respectively. The viral RNA expression relative to the average levels of housekeeping genes LAP and RpL5 is shown as the mean +/- SD of three independent replicates. H) Number of Aag2 C3PC12 cells after sequential Dhx15 or luciferase control knockdown. Bars and whiskers represent the mean +/- SD of three independent biological replicates. In panels (E, F, and H), statistical significance was determined using unpaired two tailed t-tests (* p < 0.05, *** p < 0.0005). (TIF) [file ppat.1010694.s003.tif]

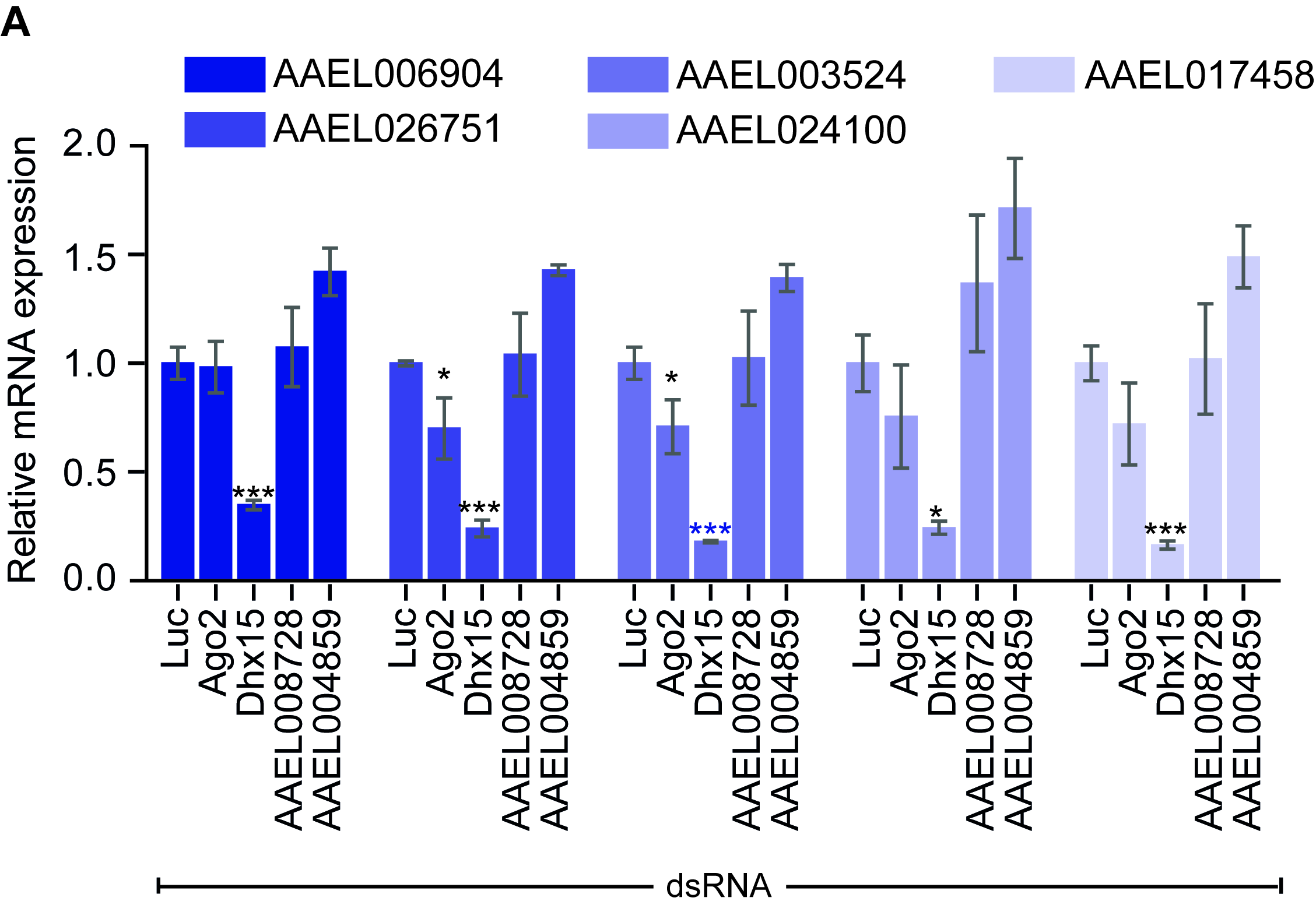

Supplement: S4 Fig — A) Quantification of top five most differentially regulated genes obtained from the RNA-sequencing list of 22 genes with shared downregulation between CHIKV infection and Dhx15 knockdown (Fig 5B). RNA levels were measured by RT-qPCR after individual silencing of Dhx15, AAEL008728, and AAEL004859 in Aag2 C3PC12 cells. Bars and whiskers represent the mean +/- SD of three independent biological replicates. Statistical significance was determined using One-Way ANOVA with Holm-Sidak correction (* p < 0.05, *** p < 0.0005). (TIF) [file ppat.1010694.s004.tif]

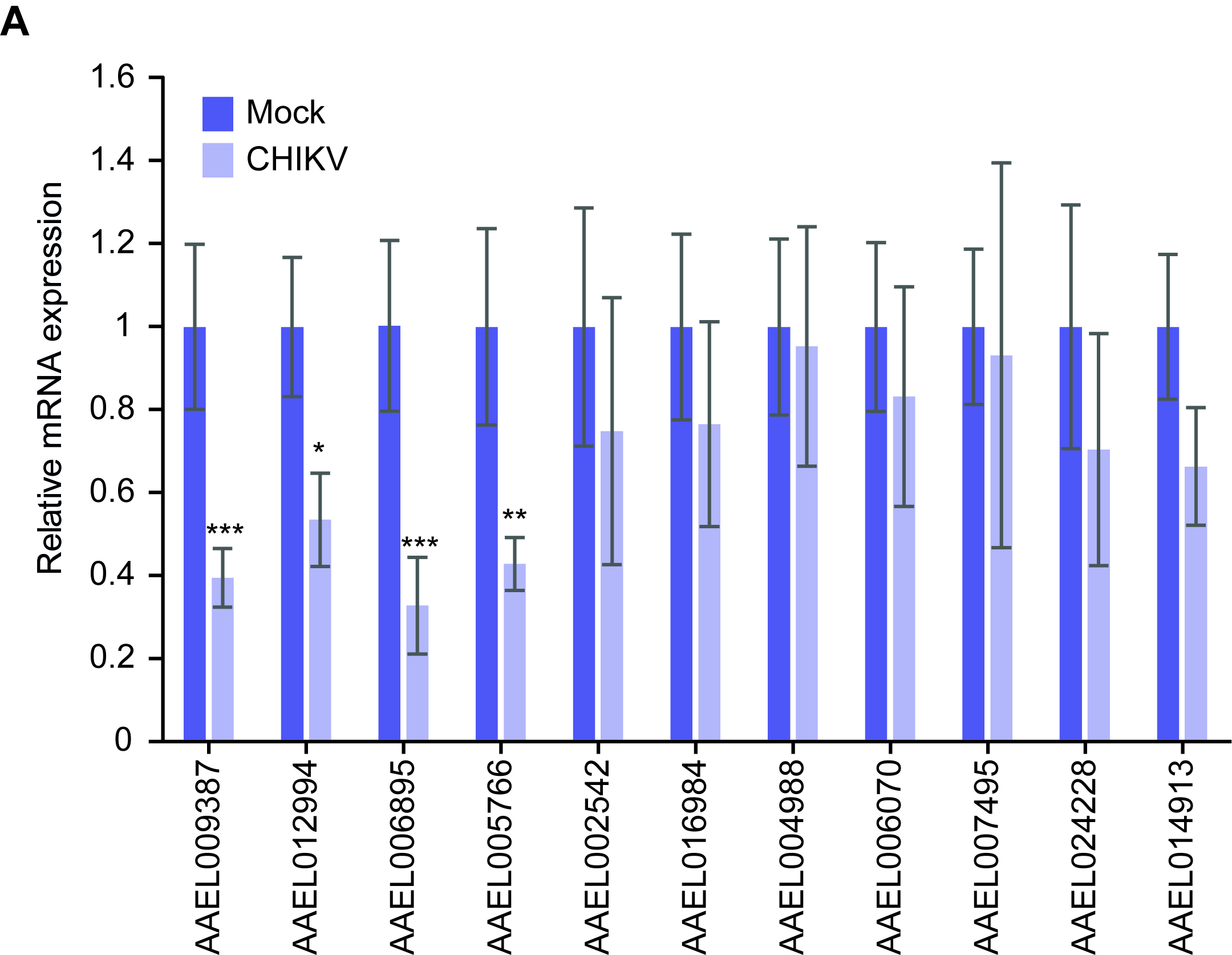

Supplement: S5 Fig — A) Relative expression of genes from the glycolysis pathway in CHIKV infected cells (MOI = 5) compared to uninfected cells in control luciferase knockdown conditions. Expression values were extracted from the RNA-sequencing data and normalized to uninfected cells. Bars and whiskers represent the mean +/- SD of three independent biological replicates. Statistics from the DESeq2 analysis are shown (* P adj < 0.05, ** P adj < 0.005, *** P adj < 0.0005). (TIF) [file ppat.1010694.s005.tif]

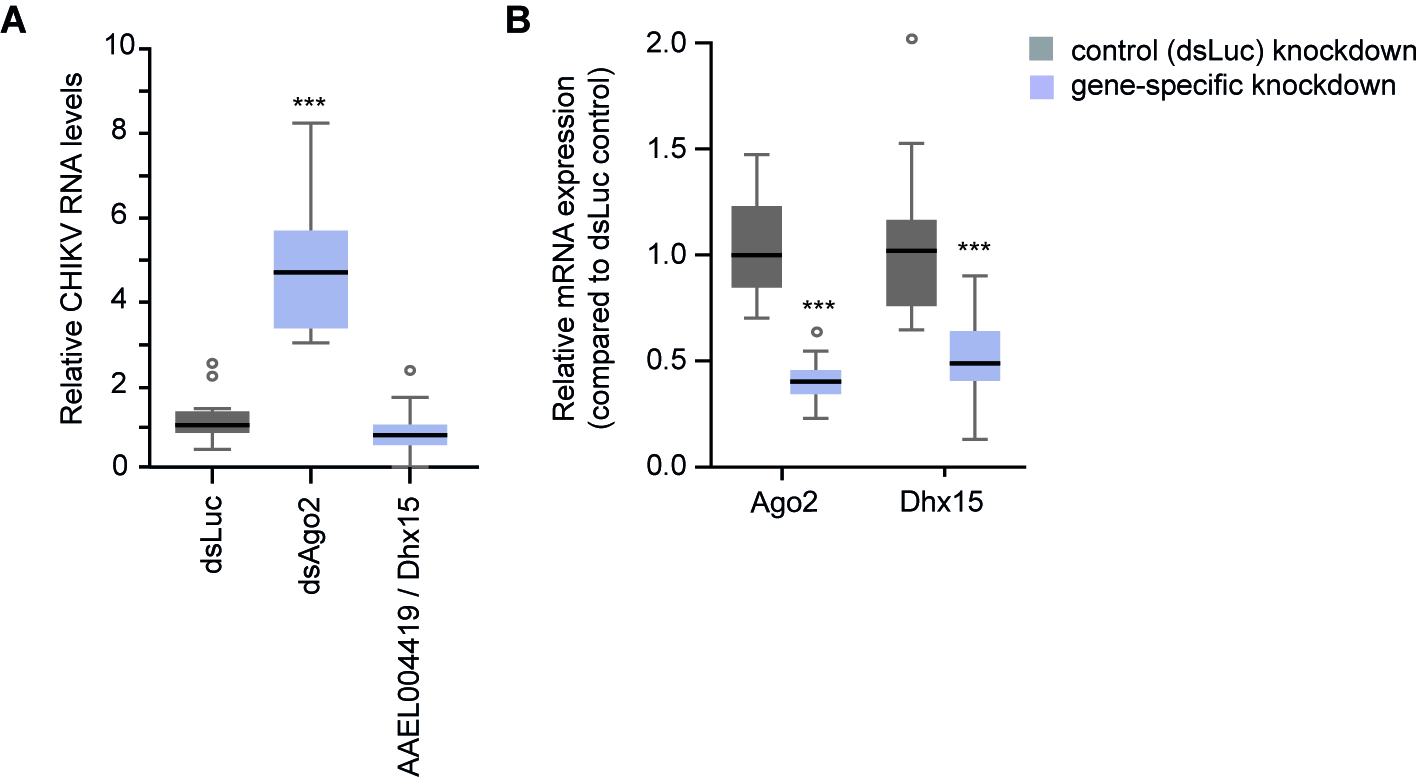

Supplement: S6 Fig — A) CHIKV replication in individual adult Ae. aegypti mosquitoes was assessed after in vivo knockdown of Dhx15 and Ago2. Viral RNA levels were quantified by RT-qPCR and normalized to the expression of LAP. Relative expression compared to virus levels in dsLuc-injected control mosquitoes is plotted. B) Knockdown efficiency was assessed in individual mosquitoes. Ago2 and Dhx15 mRNA expression was normalized against LAP expression and compared to the mean expression in the dsLuc control condition. Boxplots in (A) and (B) show median, interquartile range and maximum/minimum values. Outliers are indicated as individual dots and statistical significance was determined using unpaired two tailed t-test (*** p < 0.0005). The plot was generated in IBM SPSS v25. (TIF) [file ppat.1010694.s006.tif]
