# Supplementary material for: The DEAD-box RNA helicase Dhx15 controls glycolysis and arbovirus replication in Aedes aegypti mosquito cells
Source: PLoS Pathog. 2022 Nov 28;18(11):e1010694. doi: 10.1371/journal.ppat.1010694 (PMC9731432; doi:10.1371/journal.ppat.1010694)
Supplement: Attachment — Submitted filename: point-by-point response.pdf [file ppat.1010694.s011.pdf]

Dear editor,

Thank you for the constructive review of our manuscript entitled "The DEAD-box RNA helicase Dhx15 controls glycolysis and arbovirus replication in *Aedes aegypti* mosquito cells" written by Samara Rosendo Machado et al. and the invitation to submit a revised version of the paper. We would also especially like to thank the three reviewers for their valuable feedback. Their time and expert opinion is highly appreciated. Below please find a point-by-point response to the editorial and reviewers' comments (sorted by reviewer). Based on the feedback, we have included additional experimental evidence or adapted the text of the manuscript.

As requested, we have uploaded two versions of the manuscript, one of which highlights all changes made in response to the reviewers' comments as 'track changes'.

Editorial comment:

Comment: *We would ask specifically that the authors look into point 2 (major revision) by reviewer 3 on the limitations of the cell titre glo assay, and address this.*

Response: The reviewer highlights a valid point stating that using an ATP-based measure of cell survival may be hard to interpret when assessing silencing of genes that are involved in energy metabolism. As proposed by the reviewer, we had therefore performed direct cell counting as an additional control for cell viability when we analyzed the effect of *Dhx15* knockdown on lactate levels. These cell counts were included in our original manuscript in Figure S3G (Figure S3H in the revised version). We have rephrased the corresponding text in the results section to describe this analysis more explicitly (lines 429-431). As proposed by the reviewer, and to provide extra evidence that Dhx15 does not affect cell viability, we have also assessed the expression of a housekeeping gene, upon *Dhx15* silencing. We extracted these expression data from all knockdown experiments that were presented in the manuscript and show that Dhx15 does not significantly affect housekeeping gene expression, supporting our conclusion that cell viability is not affected (Rebuttal Figure 1).

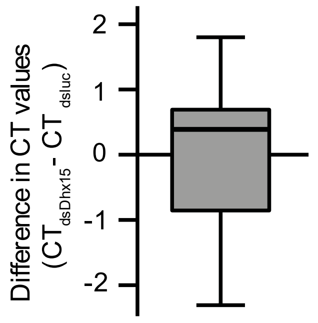

Fig.1 Effect of *Dhx15* knockdown on housekeeping gene expression. CT values for the housekeeping gene Lysosomal aspartic protease (LAP) were extracted from all qPCR data for control (dsLuc) and *Dhx15* knockdown conditions and the mean difference (ΔCT) between those conditions was calculated for each experiment. Little deviation of the ΔCT from zero (mean: 0.0075; median: 0.39) indicates similar housekeeping gene expression in control and knockdown conditions. The boxplot and whiskers show median, interquartile range and minimum/ maximum values. One sample t-test confirmed no significant difference of the mean of ΔCTs from zero (p = 0.989). Plot was generated in IBM SPSS v25.

Reviewer #1

Comment 1: *A possible weakness is the study centered CHIKV with only several accounts of using DENV. In addition, the functional experiments involving Dhx15 could have been explored further with other arboviruses. [...] Perhaps, these aspects can be investigated in the future.*

Response: Our manuscript explores the role of Dhx15 in antiviral responses against several arboviruses including Sindbis, Chikungunya and dengue virus. Knockdown of *Dhx15* significantly enhances the replication of all of these viruses, suggesting a broad antiviral activity. Importantly, Dhx15

regulates a glycolytic response also in uninfected cells (see Figure 3), indicating that the metabolic function of Dhx15 is a general mechanism, irrespective of virus infection. As suggested by the reviewer, it may in the future be interesting to functionally assess the effect of infection with arboviruses from different families on the glycolytic response. To acknowledge the valid point raised, we have elaborated our discussion on the broad antiviral role of Dhx15 beyond alphavirus infections in the revised manuscript (lines: 514-521).

Comment 2: *The establishment of Dhx15 over-expressing mosquito cells could also be a possible. Perhaps, these aspects can be investigated in the future.*

Response: We thank the reviewer for this useful suggestion. We have performed the proposed experiment and indeed found that overexpression of Dhx15, as expected, reduced replication of the SINV reporter virus. The data has now been included in Figure S2C and the text has been adapted to describe the new results and materials and methods (lines 201-206; 259-265; 382-383).

Comment 3a: *Experiments utilized different mosquito cell lines throughout the study. How come? Did persistently infected cells in some assays affected the results?*

Response: As the reviewer notes correctly, we have performed some experiments in a parental Aag2 cells and others in the Aag2-derived C3PC12 cells that were cleared from three persistently infecting viruses. This choice was not based on observations that the persistently infecting viruses affected the obtained results. Rather, Aag2 C3PC12 cells were generated in our laboratory only after the initial set of experiments, including the entire RBP screen and the first biochemical assays had already been performed. Yet, we deemed it particularly beneficial to use virus free cells for assessing transcriptomic responses. We reasoned that the presence of persistently infecting viruses may affect gene expression, possibly veiling responses induced by the arbovirus infection. Therefore, we decided to switch to the Aag2 C3PC12 cell model for the transcriptomics and all subsequent functional analysis assessing the role of Dhx15 in glycolysis. Importantly, we confirmed that the antiviral phenotype is reproducible between the two cell lines (Figure 1 and S1).

Comment 3b*: Were you able to determine the transfection efficiency of the dsRNA (single versus double transfections)?*

Response: The protocol for dsRNA transfection of Aag2 cells, including the sequential knockdown strategy to enhance knockdown efficiency, was established more than 10 years ago (Rebuttal Figure 2). Since then, it has become a routine procedure in our laboratory. We have not deviated from this protocol when we implemented Aag2 C3P12 cells and have obtained knockdown efficiencies that are as efficient as in parental Aag2 cells, strongly suggesting that dsRNA transfection efficiency is highly comparable.

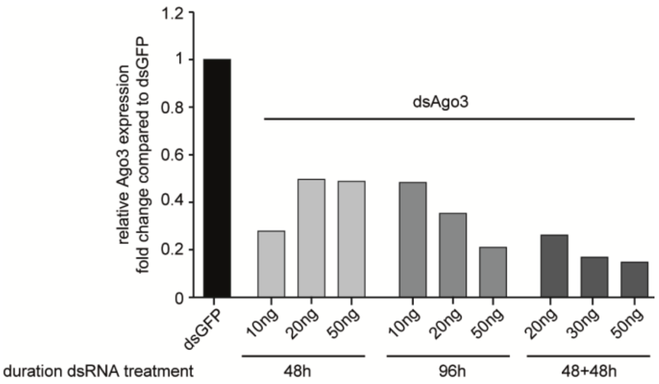

Fig.2 Optimization of dsRNA treatment of Aag2 cells.
Different dsRNA treatment regimens were compared to achieve optimal knockdown for the Ago3 gene. The knockdown strategy used in this manuscript corresponds to the 50ng sequential (48h + 48h) condition shown in the graph and has become the routine condition for knockdown in Aag2 cells in our laboratory (first described in Miesen et al., *Nucleic acids research* 2015). Plot was generated with Microsoft Excel.

***Comment 3c**: In addition, since the RBPs were cloned, knockdown efficiency could also be checked through Western blot.*

<u>Response:</u> The proposed experiment is in principle possible, but is unlikely to provide an informative assessment of knockdown efficiency, that can be extrapolated to endogenous expression of the Dhx15 protein. The expression kinetics of Dhx15 under control of its endogenous promotor versus the transgene that is driven by a poly ubiquitin promotor is likely to be very different. To illustrate this: co-transfection of dsRNA to silence ubiquitin-driven transgenes such as GFP or luciferase reporters - an experimental setup that we often use to measure the efficiency of RNA interference - works extremely efficient and reaches knockdown levels (>95%) that we rarely observe for endogenous genes. We therefore doubt that measuring knockdown efficiency by western blot using expression of a cloned transgene as readout will provide valuable additional information on knockdown efficiency of endogenous Dhx15 protein.

***Comment 4a:** Since you established the key domains of Ae. aegypti Dhx15, perhaps the paper could benefit from a structural model since existing model of the human Dhx15 are available.*

<u>Response:</u> The domains that we established in the model shown in Figure 2A are based on a prediction using the SMART database. Considering the high homology between *Aedes aegypti* and human Dhx15, we agree that generating a structural model is probably straightforward. While we appreciate this idea, we feel that it may blur the flow of the story line since no additional structural work is presented in the paper nor do we address specific questions for which the analysis of the protein structure can provide beneficial additional information. We are hesitant to solely show a structural model in the manuscript without having a concrete biological context in which to discuss this.

***Comment 4b:** In your co-IP assays you hypothesized that there could be an RNA-independent interaction between the helicases, were able to test if this is true in the presence of CHIKV viral RNA?*

<u>Response:</u> We have performed all Co-IP experiments presented in the paper in uninfected cells and indeed show that treating samples with RNases does not disturb the interaction between RNA helicases. The comment of the reviewer, to probe this interaction in CHIKV infected cells seems to imply that the presence of viral RNA should disrupt the interaction between RNA helicases. While we cannot formally exclude a scenario in which the CHIKV viral RNA serves as a scavenger to compete with protein-protein interactions, this is likely not a relevant mechanism in the context of our study. In Figure 4D, we show that the Dhx15-interacting DEAD box helicases AAEL004859 and AAEL008728 are not involved in regulating the expression of glycolytic genes. Importantly, also this analysis was performed in uninfected cells. We therefore propose that downregulation of glycolytic genes is fully independent of the protein-protein interactions of these three RNA helicases. Dhx15 thus seems to be active in multiple molecular complexes each of which may have differential functionality. We have revisited the corresponding results section to phrase our reasoning more explicit (see lines 449-451) and included an additional statement on this topic in the discussion of the revised manuscript (see lines 506-512).

***Comment 5a**: You have established that repression of Dhx15 and in turn glycolysis favors CHIKV infection. However in most cases, viral infection demands a high level of energy production and consumption in cells. Does over-expression Dhx15 in mosquito cells favor arbovirus replication?*

<u>Response</u>: Overexpression of *Dhx15* results in decreased levels of our SINV reporter in line with our previously reported observation that *Dhx15* knockdown enhances virus replication (see also

our response to comment 2). We speculate that alterations in Dhx15 shift the availability of glucose between energy metabolism and synthesis of ribonucleotides via the pentose phosphate pathway, as has been proposed for Zika virus. For a more elaborate explanation of this working model, we refer to our response to the next comment.

Comment 5b*: It may also be interesting to identify possible salvage mechanisms that may normalize gylcolysis and allow persistent infection of arboviruses in the absence of this pathway. In your transcriptomics data, were there upregulated genes that interacted with or salvaged the role of Dhx15 during glycolysis? Perhaps you can revisit your data to check for functionally redundant genes that are important and may rescue this mechanism.*

Response: The reviewer raises an interesting point about how cell survival and virus replication can be supported in an environment with presumably lowered levels of available energy (ATP) due to reduced rate of glycolysis. It should be emphasized that our readout for reduced glycolysis is production of lactate, not ATP. As indicated by the reviewer, different metabolic pathways may compensate the reduced expression of glycolytic enzymes. Interestingly, Cell titre glo assays, that we have used to assess cell viability, consistently show a slight elevation of ATP levels upon *Dhx15* knockdown, supporting the idea of a compensatory mechanism. While we do not see significant upregulation of gene expression for known genes from canonical pathways that act in energy metabolism, it is possible that these types of pathways are activated post-transcriptionally for example through post-translational modifications of metabolic enzymes.

We speculate that increased arbovirus levels upon *Dhx15* knockdown is explained by a shift of glucose availability from energy metabolism to biosynthesis of glycolysis-dependent biomolecules needed for efficient RNA replication. A strong downregulation of the rate limiting glycolytic enzyme phosphofructokinase is expected to liberate glucose that is not being metabolized to pyruvate and lactate anymore for other purposes. Upstream of phosphofructokinase, glucose enters ribonucleotide production via the pentose phosphate pathway which may support the higher demand of rNTPs during viral RNA replication. Interestingly, in mosquito cells, Zika virus infection leads to a shift of glucose fluxes from energy production via the TCA cycle to 5 carbon sugar production via the pentose phosphate pathway, underscoring the relevance of shifting the balance of glucose distribution during virus infections (Thaker *et al*., Cell Metab. 2019). In the revised manuscript, we have elaborated more on this proposed mechanism in lines 537-550.

Comment 6: *Some inconsistencies in the writing style, formatting, and abbreviations could be addressed.*

Response: We have carefully re-read the manuscript and adapted stylistic flaws and inconsistent formatting.

Reviewer #2

Comment 1*: Downregulation of phosphofructokinase, aldolase and hexokinase was identified at the transcriptional level after infection with CHIKV/knockdown of Dhx15. Would it be possible to address this at the protein level?*

Response: While we acknowledge the importance of testing knockdown efficiencies at the protein level, there are currently no mosquito antibodies available that would allow us to assess the protein expression of phosphofructokinase, aldolase and hexokinase. The amino acid sequence of these three proteins is too divergent between mosquito and human/mouse, making it highly unlikely that

mammalian antibodies would be cross-reactive. We identified two publications that describe antibodies that target *Drosophila* aldolase and hexokinase, respectively that we could have tested for cross-reactivity. Unfortunately, we were unable in obtaining these antibodies.
Importantly, in our manuscript, we show that a transcriptional downregulation of glycolytic genes has a functional consequence in reducing lactate levels. While we cannot provide a direct readout for protein expression, this functional connection between reduced glycolytic gene expression and glycolysis rate strongly imply that the level of glycolytic proteins is reduced, as well.

Comment 2*: The switch in medium to Schneider`s medium elevated baseline lactate levels. Downmodulation of glycolytic activity occurred after knockdown of Dhx15. This was discussed to generate a favorable cellular state for CHIKV infection. Thus, infection with CHIKV could be altered in the presence of Schneider`s medium. Do the authors have any indication on virus growth in Aag2 cells during altered substrate supplementation?*

Response: The use of Schneider's medium allowed us to sensitize the lactate assay, as the absence of pyruvate in combination with higher glucose levels were expected to increase glycolytic rate. We found that in Schneider's medium, the early phase of CHIKV replication is accelerated. These findings make sense in light of our hypothesis on the role of five-carbon sugar production via the pentose phosphate pathway (see also our response to comment 5b of reviewer 1). The high glucose content of Schneider's medium is expected to increase production of five-carbon sugars for nucleotide production, which we propose in our manuscript to enhance virus replication. This effect is expected to be particularly relevant in the early growth phase. Eventually, the CHIKV levels in Aag2 cells reached a similar plateau, irrespective of the medium used. We have included the growth curve of CHIKV in L15 vs Schneider's media in Figure S3G (data description in lines 421-423) and discuss the results in lines 545-547 of the revised manuscript.

Comment 3: *The authors might consider to include the following publication in their discussion: Weng, SC., Tsao, PN. & Shiao, SH. Blood glucose promotes dengue virus infection in the mosquito Aedes aegypti. Parasites Vectors 14, 376 (2021). https://doi.org/10.1186/s13071-021-04877-1. The outlined contribution of glucose to DENV infection supports the relevance of glucose/glycolysis in the context of a virus infection in mosquitoes.*

Response: We thank the reviewer for the useful suggestion. We discuss this publication in lines 551-552 of the revised manuscript.

Reviewer #3:

Comment 1a*: There are two questions that are raised by the study and require additional discussions. It is not clear how Dhx15 regulates glycolysis. The authors suggest that it is through regulation of precursors but they do not provide supportive discussion based on literature for this.*

Response: We do not share the reviewer's opinion that our manuscript does not provide information on how Dhx15 regulates glycolysis. Knockdown of *Dhx15* results in the downregulation of gene expression of core glycolytic enzymes, amongst which phosphofructokinase which mediates the committed step for glucose to be metabolized by the glycolysis pathway. It is expected that this reduced expression decreases the levels of glycolytic products, which we have verified by showing a decrease in lactate levels. It was beyond the scope of this study to further delineate the signaling cascades that link Dhx15 activity to an altered transcriptional response of glycolytic genes. We had already mentioned this open question in our initial submission (line 532-533 of the revised manuscript).

We feel that there is a misconception concerning the "regulation of precursors". We propose that regulation of metabolic precursors of nucleotides may explain how reduced glycolysis leads to enhanced virus replication (see also next comment) not how Dhx15 regulates glycolysis.

Comment 1b: *It is not clear how glycolysis inhibition reduces CHIKV and other flavivirus infections. My understanding was that glycolysis produces ATP that is required for viral replication. So a reduction in glycolysis should reduce viral replication. The authors should provide further insights here. While the study clearly identified a new antiviral factors through a screening effort that should be praised, its mechanistic characterization is not complete.*

Response: We agree that the mechanistic explanation of how reduced rates of glycolysis eventually affect virus replication is incomplete. We had acknowledged this limitation of the study in our initial submission (line 535 in the revised manuscript). We have now described in more detail, our working model to explain how reduced levels of glycolysis can increase virus replication and discussed this more extensively in light of the relevant literature (lines 537-550, See also our response to comment 5b of reviewer 1).

Comment 2: *It is known that cellular models do not always mimic the in vivo results. Authors should test the effect of Dhx15 in mosquitoes to validate their new antiviral factor.*

Response: To address the reviewer's comment we have performed an *in vivo* knockdown experiment, in which we injected dsRNA targeting Dhx15 (and Ago2 as positive control) intrathoracically. Two days later, we injected CHIKV and assessed virus replication. While this procedure results in an increase in CHIKV RNA levels for *Ago2* knockdown, we do not see an increase in virus replication after silencing *Dhx15* (Rebuttal Figure 3A). We attribute this lack of phenotype to the moderate knockdown efficiency (of about 50%; Rebuttal Figure 3B) that could be reached *in vivo*. In Aag2 cells, we regularly reach *Dhx15* knockdown of >75% and it is plausible that too high residual protein activity veils the antiviral phenotype *in vivo*. For Ago2, we obtain approximately 60% knockdown efficiency (Rebuttal Figure 3B), which is in the same order of magnitude as in our cell culture model. We have mentioned the need for an *in vivo* validation of our findings in lines 553-554.

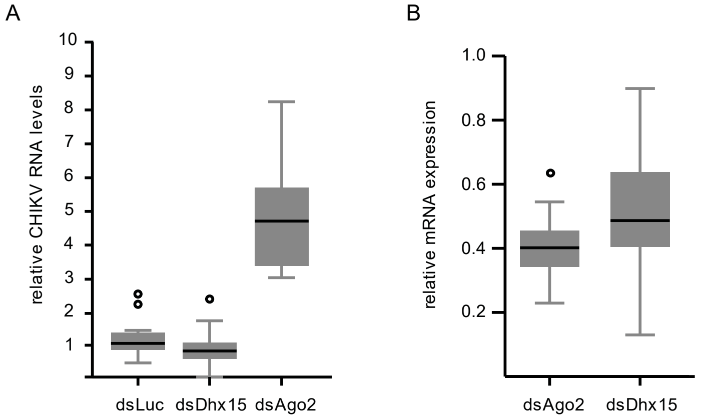

Fig.3 *Dhx15* knockdown *in vivo*. A CHIKV replication was assessed by RT-qPCR and normalized to housekeeping gene (LAP) expression. Relative expression compared to the dsLuc negative control is plotted. **B** Knockdown efficiency was assessed in individual mosquitoes. *Ago2* and *Dhx15* mRNA expression was internally normalized against *LAP* expression and normalized against the mean expression in the dsLuc control condition. Boxplots in A and B show median, interquartile range and maximum/minimum values. Outliers are indicated as individual dots. The plot was generated in IBM SPSS v25.

Comment 3*: The authors used the CellTiter-Glo kit to quantify cell survival. However this assay is based on ATP measurement, which is influenced by glycolysis, which they claim to modulate with Dxh15 kd. The authors should repeat the cell survival quantification with another assay such as cell counting or house-keeping gene quantification.*

Response: This concern was highlighted by the editor. Please see our response to the editorial comment.

Comment 4*: Using a SINV replicon for their screen, the authors missed all effects on virus assembly. This should be mentioned.*

Response: In fact, the screen was performed with an infectious virus, not a replicon. We realize that the use of a luciferase transgene incorporated in the viral genomic RNA may imply to many readers that the experiments were performed with a replicon. We have therefore re-emphasized the use of an infectious clone when introducing this experimental model in line 140 (materials and methods) and 328 (results) as well as in the legends to Figure 1A (line 594).

Comment 5: *L. 55. They reference a paper from 1983 to argue that a good understanding of mosquito factors that regulate infection is lacking. They should use a more updated review.*

Response: We have replaced this reference with more recent reviews (see line 70).

Comment 6: *L. 106. Please detail how the RBP domains were identified.*

Response: The presence of RNA binding protein domains was checked in the 'protein features and properties' annotation in VectorBase. We have specified this in the manuscript in lines 120-121.

Comment 7*: L. 319. I was surprised to see that Ago2 kd had a moderate effect as compared to other RBPs. Did the authors ensured that the Ago2 kd was sustained through the infection?*

Response: In our hands, knockdown of *Ago2* as a positive control resulted indeed in relatively moderate, yet reproducible increase of virus infection. Where RNA replication was assessed as a readout, the knockdown of gene expression was verified. For Ago2, this resulted in approximately 60% knockdown efficiency at the end of the time course of infection.

Comment 8: *L. 326. Mention the classes of the other hits, beside from the DED-box RNA helicases.*

Response: As suggested by the reviewer, we have specified the predicted protein functions of the other hits picked up by our screen (see lines 346-350).

Comment 9: *L. 433. In the re-analyse of RNAseq, it is not clearly whether the uninfected and CHIKV infected dataset were both transfected with dsRNA control.*

Response: Indeed, both datasets were treated with dsRNA. We have rephrased the sentence to clarify (see line 457).

Comment 10: *L. 437. What was the data used for Chi-square? I guess it was between the expression levels of the conditions?*

Response: The Chi-square test was done on the *number* of differentially expressed genes in the different experimental setups (see result section line 461). We aimed to statistically test, whether it would be expected by chance that out of 51 downregulated genes upon CHIKV infection n=22 would also be downregulated upon *Dhx15* silencing. To clarify this we have more explicitly explained the statistical hypothesis in lines 663-665 of the revised manuscript.

Comment 11: *L. 447-448. The last sentence of the results section is not supported by data and too speculative. It should be removed.*

Response: We have replaced this sentence with a more descriptive statement that summarizes the last part of the result section (see lines 470-471)

Comment 12*: L. 470-474. It is honest for the authors to declare the weaknesses of their study.*

Response: We agree with the reviewer that an open reflection of possible weaknesses of an experimental study is crucial for science communication. In this spirit, we have in multiple instances indicated technical or conceptual limitations of our work, some of which are inherent to RNAi screening approaches or working in non-model organisms with limited technical resources and tools (see lines 492-493, 532-534, 535, 553-554). In the course of addressing other comments of the reviewers, we have further elaborated on many of these aspects and are confident that the review process has helped in better communicating the open questions of our study.

Comment 13*: L. 501. Comparison with human Dhx15 is questionable as Dhx15 deletion did not regulate Toll-compounds or NfkB factors.*

Response: The point raised by the reviewer is very valid. In this part of the discussion we wanted to argue that it is possible that canonical immune pathways may be involved in transducing the signal from Dhx15 to eventually alteration of glycolytic gene expression. This argument was inspired by studies in mammalian system that link canonical immune signaling to glycolytic responses. We realize that discussing a study that describes activation of NFkB signaling by human Dhx15 was not a good choice to support our reasoning, as we do not find a similar response of NFkB dependent genes in mosquito cells. We have removed this part of the discussion and highlighted the open question instead (see lines 533-534).

Comment 14: *Fig. 1D and G. Please precise whether the y-axes are log or normal scales. It is confusing when comparing with 1C.*

Response: We have now explicitly stated the type of scale used per panel in the figure legends.
