# Supplementary material for: The DEAD-box RNA helicase Dhx15 controls glycolysis and arbovirus replication in Aedes aegypti mosquito cells
Source: PLoS Pathog. 2022 Nov 28;18(11):e1010694. doi: 10.1371/journal.ppat.1010694 (PMC9731432; doi:10.1371/journal.ppat.1010694)
Supplement: Attachment — Submitted filename: point-by-point response_rev.pdf [file ppat.1010694.s012.pdf]

Response to the Reviewer

Thank you for the positive feedback on our revised manuscript "*The DEAD-box RNA helicase Dhx15 controls glycolysis and arbovirus replication in Aedes aegypti mosquito cells*", written by Samara Rosendo Machado et al. Below find our response to the final reviewer's comment:

Reviewer #3

Comment: The authors should add the new in vivo data in the result section.

Response: We have included the in vivo data in figure S6 and adapted the text of the manuscript accordingly.
